# Identification of Independent Risk Factors for Skin Complications in a Multifactorial Logistic Regression Analysis of Simultaneous Immediate Autologous Breast Reconstruction and Skin Reduction Mastectomy in Large and Ptotic Breasts Using an Inferiorly Based Deepithelialized Dermal Breast Flap

**DOI:** 10.3390/jpm12030332

**Published:** 2022-02-23

**Authors:** Felix H. Vollbach, Benjamin F. Thomas, Hisham Fansa

**Affiliations:** 1 Department of Hand, Plastic and Reconstructive Surgery, Burn Center, BG Trauma Center Ludwigshafen, Ludwig-Guttmann-Strasse 13, 67071 Ludwigshafen, Germany; felix.vollbach@bgu-ludwigshafen.de (F.H.V.); benjamin.thomas@bgu-ludwigshafen.de (B.F.T.); 2 Department of Hand and Plastic Surgery, University of Heidelberg, 69117 Heidelberg, Germany; 3 Department of Plastic Surgery and Breast Center, Spital Zollikerberg, 8125 Zollikerberg, Switzerland; 4 Department of Plastic, Reconstructive, and Aesthetic Surgery, Hand Surgery, Klinikum Bielefeld, OWL-University, 33604 Bielefeld, Germany

**Keywords:** autologous breast reconstruction, DIEP-flap, thigh flap, large and ptotic breasts, inferiorly based deepithelialized flap, autoderm, independent risks, BMI, operation time, internal mammary artery perforators

## Abstract

Autologous immediate breast reconstruction in large and ptotic breasts remains challenging. We aimed to identify independent risk factors for impaired wound healing and nipple necrosis after skin reducing wise pattern mastectomy in autologous reconstruction with an auxiliary deepithelialized inferiorly based dermal flap (IBDF). Methods. This retrospective study examined patients with wise pattern mastectomy with autologous immediate breast reconstruction (IBR) between 2017 and 2019. All cases of large and ptotic breasts were included. Demographic, oncologic, reconstructive, and surgical data were compiled, and multifactorial binary logistic regression models identified independent predictors for skin complications and nipple areolar complex (NAC) necrosis. Results. Of 591 autologous breast reconstructions, 62 (11%) met the inclusion criteria. Overall wound complication rate was 32% (*n* = 20, DIEP 11, thigh 9, *p* = 0.99), including 26% minor (*n* = 16, non-surgically treated) and 7% major complications (*n* = 4, surgically treated). Complete NAC necrosis occurred in one case. Nipple sparing mastectomy (NSM) (*p* = 0.003), high BMI (*p* = 0.019), longer operation time (*p* = 0.044) and higher patient age (*p* = 0.045) were independent risk factors for skin complications. Using internal mammary artery perforators (IMAP) as recipient vessels did not result in increased complication rates (*p* = 0.59). Conclusion. Higher patient age, BMI, and operation time (OT) significantly increase the risk for skin complications in combined reduction wise pattern mastectomies with autologous IBR. In this context, IBDFs help preserve the inframammary fold, providing vasculature to the T-junction and the mastectomy skin flaps. Acceptable complication rates can be achieved in large and ptotic breasts, regardless of preoperative chemotherapy or radiation. Gentle tissue handling with minimal thermal trauma preserves internal mammary artery perforators (IMAPs) as recipient vessels. In cases of flap failure and alloplastic conversion, the IBDF can serve as an autoderm, protecting the implant from exposure

## 1. Introduction

Since obesity is an increasing phenomenon around the globe [1,2], the number of patients with large and ptotic breasts requiring surgical breast cancer treatment is consistently increasing [3]. Nipple sparing mastectomies (NSM) and skin sparing mastectomies (SSM) have both proven to be oncologically safe in prophylactic and therapeutic settings after careful oncologic and anatomic selection [4]. However, in women with large and ptotic breasts, SSM and NSM combined with immediate breast reconstruction (IBR) have higher complication rates than staged approaches [4]. Therefore, plastic surgeons usually avoid combined approaches in these patients [3]. Yet, delayed and staged techniques, cannot be applied to all cases, particularly for acute breast cancer requiring timely mastectomy. However, since impaired wound healing and skin complications after autologous IBR can significantly reduce quality of life and considerably prolong recovery time, thus delaying urgent adjuvant therapy, it potentially hampers oncological outcomes [5]. Therefore, we aimed to identify potential risk factors predicting skin complications in patients with large and ptotic breast, who underwent simultaneous autologous IBR after wise pattern SSM and NSM. In addition, we sought to highlight the potential benefit of including a deepithelialized inferiorly based dermal flap (IBDF) also known as autoderm. Thus, this study offers selection criteria for suitable patients, a better understanding of the risk factors contributing to post-operative complications, and provides guidance for improved care.

## 2. Patients and Methods

**Figure 1:**
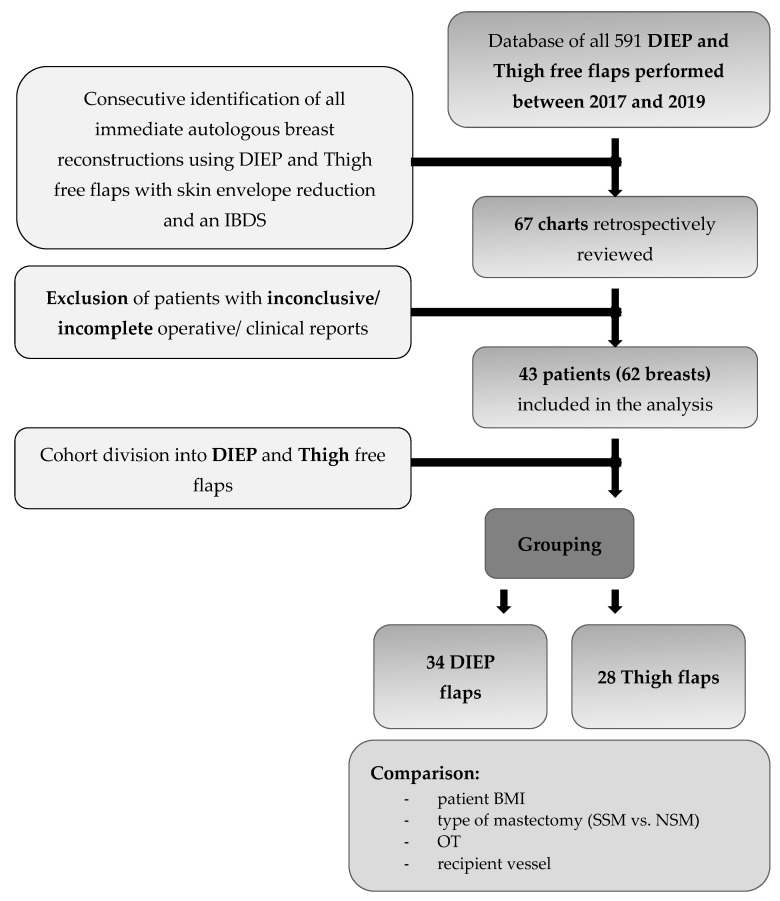
Consort Flowchart (DIEP, deep inferior epigastric artery perforator flap; Thigh, transverse myocutaneous gracilis flap or profunda artery perforator; BMI, body mass index; SSM, skin sparing mastectomy; NSM, nipple sparing mastectomy; OT, operation time).

Between 2017 and 2019, an IBDF was used for all IBR in women with large breasts and a Regnault ptosis grade 2 or 3, who underwent skin envelope reducing SSM or NSM and subsequent implant or autologous tissue-based reconstruction. We performed a retrospective monocentric chart review of potential risk factors and statistical analysis of outcomes and complications rates of all cases undergoing autologous IBR using deep inferior epigastric perforator flaps (DIEP) and inner thigh flaps (i.e., Transverse Myocutaneous Gracilis flaps (TMG), and Profunda Artery Perforator flaps (PAP)) in the senior author’s clinic. The study was performed in accordance with the ethical standards of the 1964 declaration of Helsinki and its later amendments and all patients gave written consent. Mastectomy and skin envelope reduction were performed for oncologic or prophylactic purposes by the first (F.H.V.) and senior authors (H.F.). Patients with bilateral mastectomies, or unilateral mastectomies and contralateral reduction surgery, were operated with this technique if (1) immediate autologous reconstruction was planned or (2) the original breast size was larger than the expected flap weight or (3) the patient desired breast size reduction or mastopexy (Figure 1). All reconstructions were performed by the senior author. Follow-up controls were at 3, 6, and 12-month intervals for at least 1 year post surgery.

***Markings***: Patients were preoperatively marked in the standing position using the wise pattern approach. In most cases, the distance between the nipple and the inframammary fold (IMF) exceeded 12 cm and the distance between the jugular notch and the nipple was greater than 24 cm. The upper border of the new NAC after reconstruction was planned with a 20 to 24 cm jugular notch to nipple distance, depending on the patient’s age, weight, and initial breast size. However, the distance between the nipple and the upper breast border was planned not to exceed 6–8 cm. The height of the vertical thighs (including the areola) was 10–13 cm, depending on the planned new breast size. Markings were designed more conservatively than in a classic reduction mammoplasty or mastopexy, to avoid autologous flap constriction and tension on mastectomy skin flaps. Depending on the oncological necessity and patient preference, mastectomy was performed either as a SSM or NSM. NSM was offered as an option to all women undergoing prophylactic surgery or all suitable cancer patients, dependent on cancer location, size and characteristics, after an individualized assessment by the senior author. 

**Figure 2:**
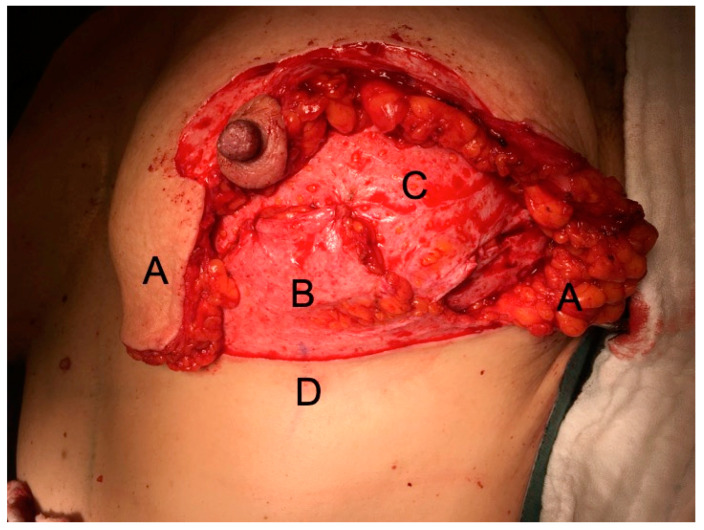
Intraoperative photograph of a DIEP flap inset after an NSM: (**A**), mastectomy skin flaps; (**B**), deepithelialized IBDF; (**C**), deepithelialized DIEP flap—the skin island to monitor flap perfusion can be positioned temporarily between the IBDF and the areola; (**D**), T-junction.

**Figure 3:**
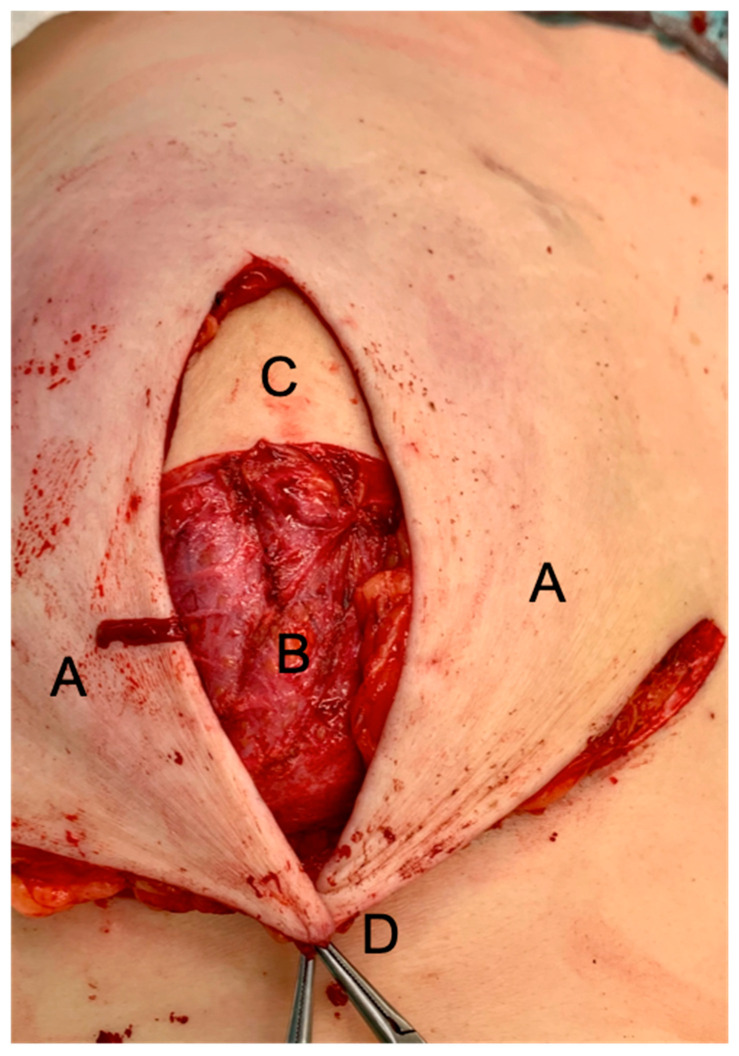
Intraoperative photograph of a DIEP flap inset after an SSM: (**A**), mastectomy skin flaps; (**B**), deepithelialized IBDF; (**C**), DIEP flap—the skin island to monitor flap perfusion can be excised later or it may serve for nipple reconstruction; (**D**), T-junction.

***Surgical technique***: Intraoperatively, the patient is placed in the supine position, with the hands positioned under the patient’s lower back and the palms facing downwards, shoulders in 30–45° flexion and elbow in 90° flexion. This allows the patient to be put in an upright position during surgery, mimicking adducted arms as much as possible. The dissection is either performed with scissors (“cold”), especially around the NAC, or with a plasma blade (Medtronic, Minneapolis, MN, USA), to avoid and reduce any thermal damage to the mastectomy skin flaps. The inferior dermis flap is deepithelialized first and then dissected from the mammary gland, carefully preserving the IMF vessels. Mastectomy is then completed. The NAC, if preserved, is dermally pedicled superio-medially. The vertical mastectomy skin flaps and the caudal dermis flap remain. Additional axillary lymph node surgery is performed without further incisions. Preferred recipient vessels are the internal mammary artery (IMA) and vein (IMV), or the internal mammary artery perforating vessels (IMAP) originating from the 2nd or 3rd intercostal space (ICS). The respective selection is made based on diameter, flow, quality, and location of the IMAP [6]. After completion of the anastomosis, the flap is sutured to the medial mastectomy border and the IMF. Then the caudal dermis flap is sutured tension-free against the flap (See Figure 2 and Figure 3). 

The dermis is completely incised caudally in the IMF, carefully preserving the perforating vessels and the subdermal plexus. The vertical thighs of the mastectomy flaps are then fixed centrally into the IMF. In NSMs, the NAC is sutured in its new position. A monitor island can be temporarily placed in the area of the vertical incision above the dermis flap (See Figure 3) and resected later if necessary. Intraoperatively, mastectomy skin and dermis flap perfusion are carefully monitored clinically, by assessing dermal bleeding from the mastectomy skin flap borders and capillary refill. If the perfusion appears insufficient intraoperatively, the mastectomy skin flaps are excised and the skin of the autologous free flap is used to replace the skin envelope. In cases where the mastectomy skin flap perfusion seemed to be compromised, deepithelization of the autologous free flap is postponed and it is set in as a “buried flap” [7]. Two to five days later, either deepithelization of the free flap or excision of the necrotic mastectomy skin flaps can be done in a controlled setting. Mastectomies were conducted at the same level for prophylactic and curative surgery, assuring to resect all glandular tissue and carefully preserving the subdermal plexus.

***Data collection***: Records were screened for patient and operative characteristics, as well as oncologic details, adjuvant therapy and postoperative complications. In detail, analyzed patient characteristics included gender, age, BMI, previous surgical and oncologic history, neo- and adjuvant therapy, BRCA status and other gene mutations, as well as comorbidities and other risk factors, such as history of smoking, allergies, and current medication. Operative characteristics included type of mastectomy (SSM, NSM), overall operation time (OT) and flap ischemia times (IT), flap type (categorized as either ‘DIEP’ or ‘Thigh’), as well as autologous flap and mastectomy specimen weights measured in grams, type and number of recipient vessels (IMA/V, IMAP), additional axillary surgery (Sentinel lymph node dissection, SLN; axillary dissection, AD) and the diameter of venous couplers used (2.0, 2.5, 3.0, or 3.5 cm). Furthermore, the incidence of breast conserving therapy (BCT), other previous surgeries, previous radiotherapy (yRX), previous chemotherapy (yCX), oncological status (categorized as ‘prophylactic mastectomy’ versus ‘DCIS’ or ‘invasive cancer’) and adjuvant selective estrogen receptor modulator (SERM) or aromatase inhibitor (AI) medication was assessed and compared between both groups. The primary endpoint assessed was the incidence of overall skin complications between both groups, defined as any impairment of skin envelope healing, ranging from epidermolysis or small wound dehiscences, manageable by either conservative wound care, or even additional surgery. The endpoint was further subcategorized into the co-secondary endpoints of either minor or major complications. Minor complications were defined as epidermolysis, impaired wound healing or wound break down, treated in an outpatient setting without additional surgery. Major complications required surgical intervention. 

***Statistical Data Analysis***: Statistics were performed with IBM SPSS Version 20 (IBM Corporation, Armonk, NY, USA) and Graphpad Prism 8 (Graphpad Software, Inc., La Jolla, CA, USA). To compare both groups regarding continuous and categorical covariates, the two-sided Welch-corrected T test, two-sided Mann–Whitney-U test and two-sided Pearson’s Chi-squared test were employed. In order to identify independent predictors of complications, a backward stepwise multifactorial binary logistic regression model was then run for each covariate, as determined by the aforementioned univariate analyses. In line with this, adjusted odds ratio (OR) estimates were reported with their respective 95% confidence intervals (CI). An error probability of *p* < 0.05 was considered statistically significant.

## 3. Results

Of all 591 autologous breast reconstructions (491 DIEP-flaps, 100 Thigh-flaps) performed between 2017 and 2019 in our institution, 62 (10.5%) met the inclusion criteria (DIEP *n* = 34, Thigh *n* = 28) and were included in this study (Figure 1). The average patient age was 45 ± 10 years (DIEP: 47 ± 12.1 years, Thigh: 43 ± 6.1 years). The average patient BMI was 25.5 ± 5.2 kg/m^2^ (DIEP: 27.9 ± 5.4 kg/m^2^; Thigh: 22.5 ± 2.7 kg/m^2^). The mean follow-up time was 14.3 ± 2.7 months. All patients were female and non-diabetic. Indications for mastectomy and breast reconstruction were for acute breast cancer (*n* = 38, 61.3%, DIEP *n* = 25, Thigh *n* = 13) and prophylactic purposes (*n* = 24, 38.7%, DIEP *n* = 9, Thigh *n* = 15). Eleven patients (18%, DIEP *n* = 2, Thigh *n* = 9) were actively smoking at the time of surgery. Two patients underwent bilateral implant and capsular removal with simultaneous SSM and free flap reconstruction (4 flaps in 2 cases, DIEP *n* = 2, Thigh *n* = 2). The mean mastectomy weight was 469 ± 215 g (DIEP: 545 ± 217.6 g; Thigh: 376 ± 170.4 g) and the mean flap weight was 439 ± 223 g (DIEP: 569 ± 212.9 g, Thigh: 280 ± 98.5 g). 22 cases received NSMs (35.5%, DIEP *n* = 11, Thigh *n* = 11) and 40 cases received SSMs (64.5%, DIEP *n* = 23, Thigh *n* = 17). Mean overall OT was 253 ± 60 min (DIEP: 258 ± 64.3 min, Thigh: 247 ± 53.9 min). For unilateral cases, the mean OT was 202 ± 44 min (DIEP: 208 ± 41.8 min, Thigh: 207 ± 45.6 min) and for bilateral cases 285 ± 44.2 min (DIEP: 302 ± 44.5 min, Thigh: 288 ± 36.7 min). The mean flap ischemia time was 40 ± 14 min (DIEP: 42 ± 14.4 min, Thigh: 38 ± 12.2 min). In 30 cases (48%, DIEP *n* = 15, Thigh *n* = 15) the IMA/V were used as recipient vessels, whereas in 32 cases (52%, DIEP *n* = 19, Thigh *n* = 13) the flaps were anastomosed to IMAPs in the 2nd or 3rd intercostal spaces (ICS) (Table 1). No revision surgery for flap congestion was needed and no flap failure occurred despite one delayed partial (DIEP) flap loss in a patient with previous BCT, yRX and yCX.

The overall skin envelope complication rate was 32% (*n* = 20, DIEP *n* = 11, Thigh *n* = 9), including 16 minor (DIEP *n* = 8, Thigh *n* = 8) and four major complications (DIEP *n* = 3, Thigh *n* = 1) (Table 2). Complete NAC necrosis occurred in one breast only (2%, Thigh *n* = 1), a smoker, who had received neoadjuvant chemotherapy up to 3 months prior to the operation and had a bilateral operation due to a BRCA mutation. Twenty-nine patients (47%, DIEP *n* = 16, Thigh *n* = 13) had chemotherapy prior to surgery and nine patients (11%, DIEP *n* = 7, Thigh *n* = 2) underwent radiation therapy before mastectomy. For further demographic details, including comorbidities and other risk factors, see Table 1, which summarizes all baseline patient and flap characteristics, as well as operative details. All patients achieved successful breast reconstruction and were satisfied with their final cosmetic results. Furthermore, no breast cancer recurrence or distant metastasis was observed during the follow-up period. Neither infections, nor hematomas, were observed at the recipient site. 

Upon univariate analysis of risk factors between patients who experienced any complication and patients without any complication, BMI (*p* = 0.026), simultaneous bilateral operations (*p* = 0.04) and increased OT (*p* = 0.003) were all significantly associated with wound complication. Furthermore, patients with NSM were more likely to develop any skin complication than patients with SSM (*p* = 0.046) (Table 3). Patients undergoing bilateral reconstruction (*p* = 0.009) and increased OT (*p* = 0.01) were significantly associated with partial NAC necrosis (Table 4). Patients who experienced impaired wound healing but required no further surgical treatment had significantly higher BMIs (24.7 ± 4.4 kg/m^2^ versus 28.1 ± 7 kg/m^2^, *p* = 0.03) (Table 5). Patients with impaired wound healing that needed revision surgeries had a significantly higher average BMI (33 ± 7 kg/m^2^ versus 24.9 ± 4.7 kg/m^2^, *p* = 0.002), received heavier flaps (739 ± 319 g versus 417.8 ± 205.3 g, *p* = 0.005), and had a significantly longer OT (312 ± 68 min versus 248.8 ± 58.5 min, *p* = 0.04) (Table 6).

Upon multifactorial binary regression analysis of independent predictors for overall incidence of any skin complications, patient BMI and the type of mastectomy (NSM vs. SSM) were significant predictors. Covariates included in the multifactorial binary regression model are listed in Table 7, along with their respective adjusted odds ratios (AOR) and 95% CIs. 

Overall, the type of mastectomy (NSM) proved to be an independent risk factor for developing skin complications, with NSMs being associated with a twofold risk increase thereof (AOR = 2.1, 95% CI: 0.3–16.1, *p* = 0.003). Moreover, every additional BMI point was associated with a 50% increase in risk of skin envelope complications or mastectomy skin flap necrosis (AOR = 1.5, 95% CI: 1.1–2.0, *p* = 0.019). Of note, heavier flaps tended to show an increased rate of skin complications (*p* = 0.066), without reaching significance. Using an IMA-Perforator (IMAP) as a recipient vessel was neither associated with higher skin envelope complications in the univariate analysis, nor upon binary regression. Upon multifactorial binary regression analysis of independent predictors for impaired wound healing addressed conservatively, overall OT (AOR = 1.2, 95% CI: 1.0–1.025, *p* = 0.44) and increasing patient age (AOR = 6.4, 95% CI 1.0–1.13, *p* = 0.045) were independent risk factors. Neither the univariate analysis, nor the multifactorial binary regression analysis, showed any negative influence on wound healing, or necrosis of NAC or skin envelope regarding axillary lymph node surgery, axillary dissection, flap size, and preoperative or post-operative chemotherapy and radiation (see Table 3, Table 4, Table 5 and Table 6).

## 4. Discussion

Breast reconstruction after skin reducing mastectomy with an autoderm flap was first described by Horton et al. in 1974 and modified later by Bostwick, who used a wise pattern mastectomy design and a deepithelialized inferiorly based dermal flap (IBDF) [8]. In principle, it resembles the caudal flap without glandular tissue in Ribeiro’s technique of reduction mammoplasty [9]. This technique is especially useful in patients with large and ptotic breasts, who desire breast volume reduction or mastopexy, accepting a smaller breast size following single-stage breast reconstruction [10]. The IBDF receives its blood supply from intercostal perforating vessels on the level of the IMF, thereby providing a vascularized layer underneath the T-junction and to the less perfused ends of the wise pattern mastectomy skin flaps. It therefore considerably mitigates hypoperfusion at the distal tips of the wise pattern mastectomy skin flaps, where most healing complications in wise pattern incisions occur [11]. 

Large autologous flaps are not always available due to a lack of donor area volume. In addition, the harvesting of large flaps may require additional surgical efforts, due to the necessity of perfusion improvement by additional pedicles (arterial/venous), thereby increasing donor site morbidity and operation time (OT). Reduced perfusion or congestion in large flaps can also cause fat necrosis. Therefore, no ubiquitously accepted agreement has yet been reached for the ideal reconstruction of large and ptotic breasts. Filling such large skin envelopes with high-volume implants does not provide a suitable solution due to increased complications and big autologous flaps are not always available [3]. 

In this context, skin or nipple sparing mastectomies demonstrate lower complication rates than skin envelope reducing techniques combined with mastectomy procedures. However, they offer less control over aesthetic aspects, such as shape, symmetry, NAC position, and breast size [4]. Delayed or staged breast reconstructions, on the other hand, sacrifice the well vascularized IBDF prior to the reconstructive procedure. 

Here, we report on our experience with an IBDF in wise pattern skin reducing SSM and NSM combined with autologous IBR. The overall complication rate of any complication observed in our study was 32.3% (*n* = 20) including 26% minor (*n* = 16, 25.8% impaired wound healing, 14.5% partial NAC necrosis) and 6.5% major (*n*= 4) complications. Of these, one was a complete NAC necrosis (1.6%) in an actively smoking patient who had received neoadjuvant chemotherapy. 

Comparing our complication rates to other studies is difficult as, to the best of our knowledge, this is the first study reporting exclusively on autologous IBR in combination with wise pattern skin reducing mastectomies with an adipocutaneous IBDF in large and ptotic breasts. All other studies focus on alternative reconstructive strategies, such as using the IBDF in skin reducing mastectomies but only implant based IBR [12,13,14], or using wise pattern incisions without the IBDF [3,4,11,15,16,17]. In a systematic review of 31 studies (1128 cases) by Tondu et al. on breast reconstruction after NSM in large and ptotic breasts, the overall complication rate was 29.1%, which is comparable to ours [4]. Of note, only four of these examine autologous IBR [3,18,19,20,21] without using an IBDF. 

Regarding studies that cover the topic of breast reconstruction in combination with an IBDF, only Rochlin et al. described skin reducing, autologous tissue IBRs in large and ptotic breasts using a deepithelialization and skin infolding technique [3]. They found slightly higher incidences for skin envelope complications with a total complication rate of 40%. Abedi et al. examined the occurrence of mastectomy flap necrosis (MFN) in 1001 IBRs in 404 patients receiving alloplastic and 314 patients receiving autologous reconstructions. They did not find significant differences between both groups and observed 24.1% mastectomy skin flap necrosis among all immediate DIEP flap reconstructions, however did not specify how many patients received a wise pattern incision [22]. Of the autologous and implant-based 151 IBRs published by Davies et al., only 28 received wise pattern mastectomies [23]. Of these, five cases (17.9%) had major complications and 11 cases (39.3%) had minor complications, corresponding to a high total complication rate of 57.7% (*n* = 16 out of 28 cases). In a prospective comparison by Lin et al. of 26 wise pattern and 27 vertical pattern mastectomies followed by autologous IBR, complications were observed in 73% of cases and 11.5% of wise pattern mastectomies required revision surgery [24]. Dec at al. analyzed 29 autologous IBR after wise pattern SSM in breasts with significant ptosis or macromastia without incorporating NSM [25]. Minor complications such as wound break down occurred in 10.3% and major complications such as mastectomy skin flap necrosis occurred in 3.4%, which is lower than in our results. Most importantly, none of the aforementioned studies used an IBDF [22,23,24,25]. 

The inferiorly based adipocutaneous flap is usually sacrificed in wise pattern mastectomies, despite representing an additional layer of well vascularized tissue and protection to the T-junction. Additionally, the inframammary fold is preserved and the flap provides support and additional volume to the lower quadrants, preventing a boxy shape of the lower pole and providing proper pocket control [12,13]. Furthermore, in cases of flap failure and alloplastic conversion, it can serve as an autoderm, protecting the implant from exposure. 

Safran et al. recently reported on 121 implant-based IBRs using an IBDF in all cases [13]. Minor and major complications were observed in 10.4% and 14.2%, respectively. When the authors compared these results to their previously published complication rates without the IBDF in immediate direct-to-implant (complication rate: 30%) and staged alloplastic reconstructions (complication rate: 20.3%), they concluded that the lower complication rate associated with the use of IBDFs could be attributed to the additional vascularized layer protecting the implant and reducing biomechanical stress on the T-junction [13]. 

Mastectomy skin flap perfusion is essential for unimpaired wound healing. In this present study, skin flap perfusion was assessed by analyzing dermal bleeding from the mastectomy skin flap edges and the corresponding capillary refill. If the perfusion seemed to be compromised, the mastectomy skin flaps were either excised and replaced by the autologous flap skin, or the flap was set in as a “buried flap” [7]. This strategy is supported by other authors as a means to preserving original breast skin and shape without the risk of uncovered flap tissue or disfigurement of the breast [7,23]. In our experience, compromised mastectomy skin envelopes display higher post-operative recovery potential in autologous breast reconstructions than in alloplastic cases. We hypothesize that this might be due to the underlying vascularized tissue, promoting neovascularization and mitigating venous congestion after a short period of vascular ingrowth. Although clinical skin flap viability assessment is most commonly used [13,24,25], skin flap perfusion mapping with laser-assisted indocyanine green (ICG) imaging has also been described [3,4,11,17,22]. Gorai et al. were able to significantly reduce postoperative full thickness mastectomy skin necrosis from 17.8% to 4.8% by using ICG intraoperatively [26]. However, ICG-VA bares the risk of allergic reactions and is not available at all surgical centers. 

In our study, higher BMI was a risk factor significantly associated with the appearance of any complication (*p* = 0.03), including wound healing disorders treated conservatively (*p* = 0.03) and surgically (*p* = 0.002). Moreover, upon multifactorial binary regression analysis, increased BMI proved to be an independent risk factor for skin envelope complications and necrosis. This was similarly observed by numerous other studies [4,19,21,23,24]. Nevertheless, we do not consider obesity a contraindication for autologous IBR in our clinic, along with other authors [27]. Significant associations between mastectomy specimen weight and complication rates described elsewhere [4,19], did not reach significance in our analysis (*p* = 0.06). Patients with NSMs were significantly more likely to develop skin and NAC complications than SSM, which is only logical as in SSM the lacking NAC offers less potential for complications. Yet, the thus far published NAC necrosis rates vary greatly (0–21.4%), with higher rates of complete NAC necrosis in immediate reconstructions compared to staged procedures (0.48% vs. 5.08%) [4]. Regardless of the pedicle type used, the NAC will always be located distant from the source of the mastectomy flaps vascularization. Therefore, longer flaps, such as in large and ptotic breasts, are more prone to impaired NAC perfusion and wound healing. In this context, high incidences of NAC necrosis in immediate implant-based breast reconstructions are caused by increased biomechanical and vascular stress on the mastectomy skin flaps, whereas autologous tissue enables neovascularization of the NAC and mastectomy skin flaps [4]. In order to address complete (1.6%) and partial (14.5%) NAC necrosis in our daily clinical practice, we now delay the NAC in an outpatient procedure under local anesthesia 10 to 14 days prior to the NSM, if oncologically possible. The principle of surgical delay can ensure NAC viability in patients at high risk [3,23,28]. The mastectomy is then either performed with scissors (“cold dissection”) or with a plasma blade (Medtronic, Minneapolis, Mn, USA), to avoid/reduce thermal damage to the mastectomy skin flaps. This was mainly due to our conviction that gentle tissue handling without any unnecessary thermal trauma will lead to the best outcome. Mastectomy performed with diathermy appears to speed up tissue dissection and coagulation intraoperatively [28]. Increased thrombosis of sub-dermal vessels caused by diathermia, which is essential for sufficient coagulation, may cause tissue ischemia. The idea of the surgical technique being responsible for high complication rates in NSM, is supported by numerous authors [15,17,18,19,20]. Among these, some use a tumescent injection [3,17,23] or propose to perform the mastectomy with minimal electrocautery [15,17,18,20] but none compared the effect on the outcome of these different techniques. In a multi-institutional analysis of 1935 NSM, the incidences of minor (10%) and major (4.5%) complications were compared to the techniques used for mastectomy (electrocautery 43.8%, plasma knife 31%, sharp dissection 11.9%, sharp dissection with tumescent injection 11.7%, unknown 1.6%). Notably sharp dissection with tumescent injection had the highest incidence (13.7%) for mastectomy skin flap infections, which was more than ten times higher than the incidence of the plasma blade (1.2%), Electrocautery (3.5%) or sharp dissection alone (7.4%) [29]. These incidences are lower than those we observed in our study, but the authors did not incorporate any wise pattern mastectomies.

In our study, bilateral reconstructions (DIEP and Thigh) were significantly more likely to develop complications (*p* ≤ 0.04), particularly partial NAC necrosis (*p* = 0.009). These cases are associated with longer operation times (OT), which was also significantly associated with the appearance of any complication (*p* = 0.003), including partial NAC necrosis (*p* = 0.01) and impaired wound healing requiring surgical treatment (*p* = 0.04). Upon multifactorial binary regression analysis, increased OT was an independent risk factor for impaired wound healing addressed conservatively with a 1.2% risk increase for every additional minute of operation (AOR = 1.02, 95% CI: 1.0–1.025, *p* = 0.044). We believe that this observation can be explained by prolonged mechanical strain on mastectomy skin flaps. In line with our observations, Abedi et al. found OT to be significantly associated with MFN upon univariate (*p* = 0.002) as well as upon multivariable logistic regression analysis (*p* = 0.001) in their analysis of 404 alloplastic and 314 autologous IBR patients [22].

Interestingly, using IMAPs from the 2nd or 3rd ICS as recipient vessels, was not associated with higher skin envelope complications in the univariate analysis. In this context, it is essential to preserve as many perforators from the ICS as possible to compensate for the reduction in skin perfusion associated with the use of IMAPs for the anastomosis [12,13,18,20,24]. In a previous study, we were able to show that IMAPs can be safely used as recipient vessels in autologous IBR even after radiation therapy [6]. In this present study, they prove to be reliable even in wise pattern SSM and NSM without increasing complication rates and without any flap failures. Prior radiation therapy is considered a contraindication for NSM by some authors due to decreased dermal vasculature and increased healing disorders [4]. Mitchell et al. observed significantly more major complications after prior radiation therapy in their analysis of 1935 NSMs without specifying the type of reconstruction [29]. We did not observe any negative effect on wound healing or skin flap necrosis neither by pre-operative nor post-operative radiation therapy in our patients, in line with the finding of others [11,17,19,21]. We hypothesize that this might be attributable to the revascularizing potential of both autologous flap and the IBDF. Furthermore, we did not observe any negative effect on wound healing or skin flap necrosis neither by pre-operative nor post-operative chemotherapy. Therefore, we consider neither radio- nor chemo-therapy contraindications for autologous IBR.

The limitations of this study lie in its design of a retrospective chart review and the limited number of patients. The decision for or against NSM might be biased and there are no aesthetic or patient reported outcomes measured.

## 5. Conclusions

Higher BMI and prolonged OT increase complication rates in autologous IBR after wise pattern SSM or NSM. Therefore, bilateral reconstructions should be conducted in separate procedures if possible (thigh-based flaps). Gentle tissue handling with minimal thermal trauma and preservation of ICS perforators maintains IMAPs as recipient vessels. NAC conditioning prior to the mastectomy procedures can help reduce complication rates. The IBDF not only helps to maintain the inferior anatomical boarder of the breast, but also provides additional vasculature to the T-junction and overlying skin flaps and can serve as a protective autoderm in cases of flap failure and alloplastic conversion.

## Figures and Tables

**Table 1:** Univariate analysis of patient, operative, and flap covariates between the DIEP and Thigh flap groups.

		Flap Types			
	Overall	DIEP	Thigh	*p*-Value	Significant
Number of cases (%)	* n * = 62 (100%)	* n * = 34 (54.8%)	*n* = 28 (45.2%)	-	-
* Patient Characteristics *	-	-	-	-	-
Mean age ± SD [years]	45.1 ± 10.0	46.9 ± 12.1	43 ± 6.1	0.1	
BMI ± SD [kg/m^2^]	25.5 ± 5.2	27.9 ± 5.4	22.5 ± 2.7	<0.0001	**
Comorbidities	-	-	-	-	-
Smoking	11 (100%)	2 (18.2%)	9 (81.8%)	0.007	**
Alcohol	7 (100%)	1 (14.3%)	6 (85.7%)	0.02	*
Diabetes	0	0	0	-	-
* Operative Characteristics *	-	-	-	-	-
Unilateral	24 (100%)	16 (66.7%)	8 (33.3%)	0.1	
Bilateral	19 (100%)	9 (47.4%)	10 (52.6%)	0.1	
SSM	40 (100%)	23 (57.5%)	17 (42.5%)	0.6	
NSM	22 (100%)	11 (50%)	11 (50%)	0.6	
Internal mammary artery/vein (IMA/V)	30 (100%)	15 (50%)	15 (50%)	0.6	
Int. mam. artery/vein perf. (IMAP)	32 (100%)	19 (59.4%)	13 (40.6%)	0.5	
Mean Coupler ± SD [mm]	2.4 ± 0.3	2.4 ± 0.3	2.3 ± 0.3	0.2	
Mean mastectomy weight ± SD [g]	468.8 ± 214.7	544.9 ± 217.6	376.4 ± 170.4	0.002	**
Mean flap weight ± SD [g]	438.5 ± 223.4	569.0 ± 212.9	280.1 ± 98.5	<0.0001	**
Mean OT ± SD [minutes] †	252.9 ± 60.1	258.1 ± 64.3	246.6 ± 53.9	0.5	
Mean OT ± SD [minutes]—unilateral	201.5 ± 43.8	208.3 ± 41.8	207.3 ± 45.6	0.3	
Mean OT ± SD [minutes]—bilateral	285.4 ± 44.2	302.4 ± 44.5	287.9 ± 36.7	0.03	*
Mean ischemia time ± SD [minutes]	39.9 ± 13.6	41.6 ± 14.4	37.7 ± 12.2	0.3	
* Adjuvant oncologic therapy *	-	-	-	-	-
Invasive cancer	28 (100%)	18 (64.3%)	10 (35.7%)	0.2	
DCIS	9 (100%)	6 (66.7%)	3 (33.3%)	0.4	
Prophylactic surgery	25 (100%)	10 (40%)	15 (60%)	0.05	*
Prev. breast conserving therapy	13 (100%)	11 (84.6%)	2 (15.4%)	0.02	*
Prev. radiation therapy (prev. Rx)	9 (100%)	7 (77.8%)	2 (22.2%)	0.1	
Prev. chemo therapy (prev. Cx)	29 (100%)	16 (55.2%)	13 (44.8%)	0.9	
Postop. Radiation therapy (post. Rx)	7 (100%)	3 (42.9%)	4 (57.1%)	0.5	
Postop. chemo therapy (post. Cx)	10 (100%)	5 (50%)	5 (50%)	0.7	
Sentinel lymph node biopsy (SLN)	24 (100%)	15 (62.5%)	9 (37.5%)	0.3	
Axillary lymph node dissection	5 (100%)	2 (40%)	3 (60%)	0.5	

* *p* < 0.05, ** *p* < 0.01, SD = Standard Deviation, † Overall operative time: includes breast pocket preparation (mastectomy, recipient vessel dissection, lymph node surgery), simultaneous flap raising, anastomosis, flap insetting and simultaneous donor site closure.

**Table 2:** Univariate Comparison of Breast-Site Complications between DIEP and Thigh groups.

		Flap Types			
	Overall	DIEP	Thigh	*p*-Value	Significant
* Number of cases (%) *	* n * = 62 (100%)	* n * = 34 (54.8%)	* n * = 28 (45.2%)	—	—
* SSM *	40 (64.5%)	23 (67.6%)	17 (60.7%)		
* NSM *	22 (35.5%)	11 (32.4%)	11 (39.2%)		
* Any complication (% within flap type) *	* n * = 20 (32.2%)	* n * = 11 (32.4%)	* n * = 9 (32.1%)	0.99	n.s.
* Minor complications (no surgery) *	—	—	—	—	—
Impaired WH breast skin	16 (25.8%)	8 (23.5%)	8 (28.6%)	>0.99	n.s.
Partial NAC necrosis	9 (14.5%)	5 (8.1%)	4 (14.3%)	0.963	n.s.
* Major complication (surg. treated) *	—	—	—	—	—
Complete flap loss	0	0	0	—	—
Partial flap loss	0	0	0	—	—
Complete NAC necrosis	1 (1.6%)	0	1 (3.6%)	0.267	n.s.
Impaired WH breast	4 (6.5%)	3 (4.8%)	1 (3.6%)	0.402	n.s.

n.s. = not significant, WH: Wound healing, these include every incidence of impaired healing.

**Table 3:** Univariate analysis of risk factors between patients with and without skin complications.

		Any Skin Complications			
	Overall	Any Complication	No Complication	*p*-Values	Significant
* Number of cases (%) *	* n * = 62 (100%)	* n * = 20 (32.3%)	*n* = 42 (67.7%)	-	-
* Patient Characteristics *					
Mean age ± SD [years]		45.7 (±11.4)	44.9 (±9.5)	0.77	n.s.
Mean BMI ± SD [kg/m^2^]		27.6 (±6.4)	24.4 (±4.2)	0.026	*
Comorbidities					
Active smoker/non-smoker (%)		4 (20%)/16 (80%)	7 (16.7%)/35 (83.3%)	0.74	n.s.
Alcohol/no Alcohol (%)		2 (10%)/18 (90%)	5 (11.9%)/37 (88.1%)	0.99	n.s.
* Operative Characteristics *					
Flap type (DIEP/Thigh)		11 (55%)/9 (45%)	23 (54.8%)/19 (45.2%)	0.99	n.s.
Unilateral/bilateral reconstruction		4 (20%)/16 (80%)	20 (47.6%)/22 (52.4%)	0.05	*
SSM/NSM		9 (45%)/11 (55%)	31 (73.8%)/11 (26.2%)	<0.05	*
Recipient Vessel (IMA/IMAP)		11 (55%)/9 (45%)	19 (45.2%)/23 (54.8%)	0.59	n.s.
Coupler rank [1.5/2.0/2.5/3.0 mm] (%)		(0/3/16/1) (0%/15%/80%/5%)	(2/13/25/2) (4.8%/31%/59.5%/4.8%)	0.37	n.s.
Mean mastectomy weight ± SD [g]		543.4 (±237)	433.3 (±199.2)	0.06	n.s.
Mean flap weight ± SD [g]		505.5 (±264.8)	406.7 (±199.3)	0.11	n.s.
Mean operative time ± SD [minutes] †		285.4 (±56.4)	237.5 (±56.8)	0.003	**
Mean ischemia time ± SD [minutes]		37.9 (±10.98)	40.8 (±14.7)	0.44	n.s.
* Adjuvant oncologic therapy *					
Invasive Cancer/no inv. Ca.		8 (40%)/12 (60%)	20 (47.6%)/22 (52.4%)	0.6	n.s.
DCIS/no DCIS (%)		3 (15%)/17 (85%)	6 (14.3%)/36 (85.7%)	0.99	n.s.
Prophylactic/non-prophylactic surgery (%)		8 (40%)/12 (60%)	14 (33.3%/28 (66.7%)	0.78	n.s.
Prev. BCT/no BCT (%)		4 (20%)/16 (80%)	9 (21.4%)/33 (78.6%)	0.99	n.s.
Prev. Rx/no Rx (%)		3 (15%)/17 (85%)	6 (14.3%)/36 (85.7%)	0.99	n.s.
Prev. Cx/no Cx (%)		8 (40%)/12 (60%)	21 (50%)/21 (50%)	0.59	n.s.
Postop. Rx (%)		1 (5%)	6 (14.3%)	0.41	n.s.
Postop. Cx (%)		2 (10%)	8 (19%)	0.48	n.s.
SLN/no SLN (%)		8 (40%)/12 (60%)	16 (38.1%)/26 (61.9%)	0.99	n.s.
ALND/no ALND (%)		0 (0%)/20 100%)	5 (11.9%)/37 (88.1%)	0.17	n.s.

* *p* < 0.05, ** *p* < 0.01, n.s. = not significant, SD = Standard Deviation, m^2^ = square meters, Ca. = Carcinoma, IMA = Internal mammary artery, IMAP = Internal mammary artery perforator, BCT = breast conserving therapy, Rx = Radiation therapy, Cx = Chemo therapy, SLN = Sentinel lymph node surgery, ALND = Axillary lymph node dissection, † Overall operative time: includes breast pocket preparation (mastectomy, recipient vessel dissection, lymph node surgery), simultaneous flap raising, anastomosis, flap insetting and simultaneous donor site closure.

**Table 4:** Univariate analysis of risk factors between cases with and without partial NAC necrosis.

		Partial NAC Necrosis			
	Overall	NAC Necrosis	No NAC Necrosis	*p*-Values	Significant
Number of cases (%)	n = 62 (100%)	n = 9 (14.5%)	n = 53 (85.5%)	-	-
* Patient Characteristics *					
Mean age ± SD [years]		38 (± 10.7)	46.4 (±9.6)	0.02	*
Mean BMI ± SD [kg/m^2^]		24.9 (± 5.5 )	25.5 (±5.2)	0.73	n.s.
Comorbidities					
Active smoker/non-smoker (%)		2 (22.2%)/7 (77.8%)	9 (17%)/44 (83%)	0.66	n.s.
Alcohol/no Alcohol (%)		2 (22.2%)/7 (77.8%)	5 (9.4%)/48 (90.6%)	0.27	n.s.
* Operative Characteristics *					
Flap type (DIEP/Thigh) (%)		5 (55.6%)/4 (44.4%)	29 (54.7%)/24 (45.3%)	0.99	n.s.
Unilateral/bilateral reconstruction (%)		0 (0%)/9 (100%)	24 (45.3%)/29 (54.7%)	0.009	**
SSM/NSM (%)		0 (0%)/9 (100%)	40 (75.5%)/13 (24.5%)	< 0.001	**
Recipient Vessel (IMA/IMAP) (%)		4 (44.4%)/5 (55.6%)	26 (49.1%)/27 (50.9%)	0.99	n.s.
Coupler rank [1.5/2.0/2.5/3.0 mm] (%)		(0/2/7/0) (0%/22.2%/77.8%/0%)	(2/14/34/3) (3.8%/26.4%/64.2%/5.7%)	0.77	n.s.
Mean mastectomy weight ± SD [g]		518.7 (± 292.9)	460 (±203.1)	0.58	n.s.
Mean flap weight ± SD [g]		492.4 (± 324.2)	429.4 (±206.7)	0.59	n.s.
Mean operative time ± SD [minutes] †		300.4 (± 43)	244.9 (±59.7)	0.01	*
Mean ischemia time ± SD [minutes]		35.4 (± 7.96)	40.6 (±14.2)	0.3	n.s.
* Adjuvant oncologic therapy *					
Invasive Cancer/no inv. Ca.		2 (22.2%)/7 (77.8%)	26 (49.1%)/27 (50.9%)	0.17	n.s.
DCIS/no DCIS (%)		2 (22.2%)/7 (77.8%)	7 (13.2%)/46 (86.8%)	0.61	n.s.
Prophylactic/non-prophylactic surgery (%)		6 (66.7%)/3 (33.3%)	16 (30.2%)/37 (69.8%)	0.057	n.s.
Prev. BCT/no BCT (%)		0 (0%)/9 (100%)	13 (24.5%)/40 (75.5%)	0.18	n.s.
Prev. Rx/no Rx (%)		1 (11.1%)/8 (88.9%)	8 (15.1%)/45 (84.9%)	0.99	n.s.
Prev. Cx/no Cx (%)		2 (22.2%)/7 (77.8%)	27 (50.9%)/26 (49.1%)	0.16	n.s.
Postop. Rx (%)		0 (0%)	7 (13.2%)	0.58	n.s.
Postop. Cx (%)		1 (11.1%)	9 (17%)	0.99	n.s.
SLN/no SLN (%)		2 (22.2%)/7 (77.8%)	22 (41.5%)/31 (58.5%)	0.46	n.s.
ALND/no ALND (%)		0 (0%)/9 (100%)	5 (9.4%)/48 (90.6%)	0.99	n.s.

* *p* < 0.05, ** *p* < 0.01, n.s. = not significant, SD = Standard Deviation, m^2^ = square meters, Ca. = Carcinoma, IMA = Internal mammary artery, IMAP = Internal mammary artery perforator, BCT = breast conserving therapy, Rx = Radiation therapy, Cx = Chemo therapy, SLN = Sentinel lymph node surgery, ALND = Axillary lymph node dissection, † Overall operative time: includes breast pocket preparation (mastectomy, recipient vessel dissection, lymph node surgery), simultaneous flap raising, anastomosis, flap insetting and simultaneous donor site closure.

**Table 5:** Univariate analysis of risk factors between cases with impaired WH treated conservatively and cases without impaired WH.

		Impaired WH Treated Conservatively			
	Overall	Impaired WH	Uneventful WH	*p*-Values	Significant
* Number of cases (%) *	* n * = 62 (100%)	* n * = 14 (22.6%)	*n* = 48 (77.4%)	-	-
* Patient Characteristics *					
Mean age ± SD [years]		49.1 ( ±10.7)	44 (±9.6)	0.09	n.s.
Mean BMI ± SD [kg/m^2^]		28.1 ( ±6.98 )	24.7 (±4.4)	0.03	*
Comorbidities					
Active smoker/non-smoker (%)		2 (14.3%)/12 (85.7%)	9 (18.8%)/39 (81.2%)	0.99	n.s.
Alcohol/no Alcohol (%)		0 (0%)/14 (100%)	7 (14.6%)/41 (85.4%)	0.33	n.s.
* Operative Characteristics *					
Flap type (DIEP/Thigh) (%)		9 (64.3%)/5 (35.7%)	25 (52.1%)/23 (47.9%)	0.55	n.s.
Unilateral/bilateral (%)		4 (28.6%)/10 (71.4%)	20 (41.7%)/28 (58.3%)	0.54	n.s.
SSM/NSM (%)		9 (64.3%)/5 (35.7%)	31 (64.6%)/17 (35.4%)	0.99	n.s.
Recipient Ves. (IMA/IMAP)(%)		8 (57.1%)/6 (42.9%)	22 (45.8%)/26 (54.2%)	0.55	n.s.
Coupler rank [1.5/2.0/2.5/3.0 mm] (%)		(0/2/11/1) (0%/14.3%/78.6%/7.1%)	(2/14/30/2) (4.2%/29.2%/62.5%/4.2%)	0.55	n.s.
Mean mastectomy weight ± SD [g]		522.9 ( ±229.2)	453.0 (±212.5)	0.29	n.s.
Mean flap weight ± SD [g]		501.6 ( ±224.6)	420.1 (±224.3)	0.24	n.s.
Mean operative time ± SD [minutes] †		274.5 ( ±60.4)	246.7 (±59.8)	0.13	n.s.
Mean ischemia time ± SD [minutes]		39.8 ( ±11.4)	39.9 (±14.3)	0.98	n.s.
* oncologic characteristics *					
Invasive Cancer/no inv. Ca.		7 (50%)/7 (50%)	21 (43.8%)/27 (56.2%)	0.77	n.s.
DCIS/no DCIS (%)		2 (14.3%)/12 (85.7%)	7 (14.6%)/41 (85.4%)	0.99	n.s.
Prophylactic/non-prophylac. (%)		4 (28.6%)/10 (71.4%)	18 (37.5%)/30 (62.5%)	0.75	n.s.
Prev. BCT/no BCT (%)		4 (28.6%)/10 (71.4%)	9 (18.8%)/39 (81.2%)	0.47	n.s.
Prev. Rx/no Rx (%)		3 (21.4%)/11 (78.6%)	6 (12.5%)/42 (87.5%)	0.41	n.s.
Prev. Cx/no Cx (%)		8 (57.1%)/6 (42.9%)	23 (47.9%)/25 (52.1%)	0.77	n.s.
Postop. Rx (%)		1 (7.1%)	6 (12.5%)	0.99	n.s.
Postop. Cx (%)		1 (7.1%)	9 (18.8%)	0.43	n.s.
SLN/no SLN (%)		6 (42.9%)/8 (57.1%)	18 (37.5%)/30 (62.5%)	0.76	n.s.
ALND/no ALND (%)		0 (0%)/14 (100%)	5 (10.4%)/43 (89.6%)	0.58	n.s.

* *p* < 0.05, n.s. = not significant, SD = Standard Deviation, m^2^ = square meters, Ca. = Carcinoma, IMA = Internal mammary artery, IMAP = Internal mammary artery perforator, BCT = breast conserving therapy, Rx = Radiation therapy, Cx = Chemotherapy, SLN = Sentinel lymph node surgery, ALND = Axillary lymph node dissection, † Overall operative time: includes breast pocket preparation (mastectomy, recipient vessel dissection, lymph node surgery), simultaneous flap raising, anastomosis, flap insetting and simultaneous donor site closure.

**Table 6:** Univariate analysis of risk factors between cases with impaired wound healing treated surgically (major complications) and cases without wound healing impairments.

		Impaired WH with Surgical Treatment			
	Overall	WH Disorder	No Surgery	*p*-Values	Significant
* Number of cases (%) *	* n * = 62 (100%)	* n * = 4 (6.5%)	*n* = 58 (93.5%)	-	-
* Patient Characteristics *					
Mean age ± SD [years]		44 ( ±15.3)	45.2 (±9.8)	0.82	n.s.
Mean BMI ± SD [kg/m^2^]		33 ( ±7 )	24.9 (±4.7)	0.002	**
Comorbidities					
Active smoker/non-smoker (%)		1 (25%)/3 (75%)	10 (17.2%)/48 (82.8%)	0.55	n.s.
Alcohol/no Alcohol (%)		0 (0%)/4 (100%)	7 (12.1%)/51 (87.9%)	0.99	n.s.
* Operative Characteristics *					
Flap type (DIEP/Thigh) (%)		3 (75%)/1 (25%)	31 (53.4%)/27 (46.6%)	0.62	n.s.
Unilateral/bilateral reconstruction (%)		1 (25%)/3 (75%)	23 (39.7%)/35 (60.3%)	0.99	n.s.
SSM/NSM (%)		1 (25%)/3 (75%)	39 (67.2%)/19 (32.8%)	0.12	n.s.
Recipient Vessel (IMA/IMAP) (%)		3 (75%)/1 (25%)	27 (46.6%)/31 (53.4%)	0.35	n.s.
Coupler rank [1.5/2.0/2.5/3.0 mm] (%)		(0/0/4/0) (0%/0%/100%/0%)	(2/16/37/3) (3.4%/27.6%/63.8%/5.2%)	0.53	n.s.
Mean mastectomy weight ± SD [g]		566.5 ( ±284.2)	462.1 (±212.6)	0.36	n.s.
Mean flap weight ± SD [g]		739.0 (±319.2)	417.8 (±205.3)	0.005	**
Mean operative time ± SD [minutes] †		312.25 ( ±68.1)	248.8 (±58.5)	0.04	*
Mean ischemia time ± SD [minutes]		34.5 ( ±11.1)	40.2 (±13.7)	0.42	n.s.
* Adjuvant oncologic therapy *					
Invasive Cancer/no inv. Ca.		2 (50%)/2 (50%)	26 (44.8%)/32 (55.2%)	0.99	n.s.
DCIS/no DCIS (%)		0 (0%)/4 (100%)	9 (15.5%)/49 (84.5%)	0.99	n.s.
Prophylactic/non-prophylactic surgery (%)		1 (25%)/3 (75%)	21 (36.2%/37 (63.8%)	0.99	n.s.
Prev. BCT/no BCT (%)		1 (25%)/3 (75%)	12 (20.7%)/46 (79.3%)	0.99	n.s.
Prev. Rx/no Rx (%)		1 (25%)/3 (75%)	8 (13.8%)/50 (86.2%)	0.48	n.s.
Prev. Cx/no Cx (%)		1 (25%)/3 (75%)	26 (44.8%)/32 (55.2%)	0.33	n.s.
Postop. Rx (%)		0 (0%)	7 (12.1%)	0.99	n.s.
Postop. Cx (%)		0 (0%)	10 (17.2%)	0.99	n.s.
SLN/no SLN (%)		2 (50%)/2 (50%)	22 (37.9%)/36 (62.1%)	0.64	n.s.
ALND/no ALND (%)		0 (0%)/4 (100%)	5 (8.6%)/53 (91.4%)	0.99	n.s.

* *p* < 0.05, ** *p* < 0.01, n.s. = not significant, SD = Standard Deviation, m^2^ = square meters, Ca. = Carcinoma, IMA = Internal mammary artery, IMAP = Internal mammary artery perforator, BCT = breast conserving therapy, Rx = Radiation therapy, Cx = Chemotherapy, SLN = Sentinel lymph node surgery, ALND = Axillary lymph node dissection, † Overall operative time: includes breast pocket preparation (mastectomy, recipient vessel dissection, lymph node surgery), simultaneous flap raising, anastomosis, and flap insetting, simultaneous donor site closure.

**Table 7:** Multifactorial Binary Logistic Regression Analysis with Adjusted Odds Ratios for Independent Predictors per Dependent Variable.

Variable	Adjusted Odds Ratio	95% CI	*p*-Values	Significant
* Any Complication *				
BMI	1.48	1.06–2.05	0.019	*
Mastectomy type SSM vs. NSM	21.38	2.9–160.5	0.003	**
* Impaired wound healing treated conservatively *				
Operation time (OT)	1.01	1.00–1.03	0.044	*
Age	1.06	1.00–1.13	0.045	*

* *p* < 0.05, ** *p* < 0.01, BMI = body mass index [kg/m^2^], SSM = skin-sparing mastectomy, NSM = nipple-sparing mastectomy.

## Data Availability

Supporting data is available from the authors upon request.
